# Supplementary figures and images for: Real-time and high precision feature matching between blur aerial images
Source: PLoS One. 2022 Sep 19;17(9):e0274773. doi: 10.1371/journal.pone.0274773 (PMC9484699; doi:10.1371/journal.pone.0274773)

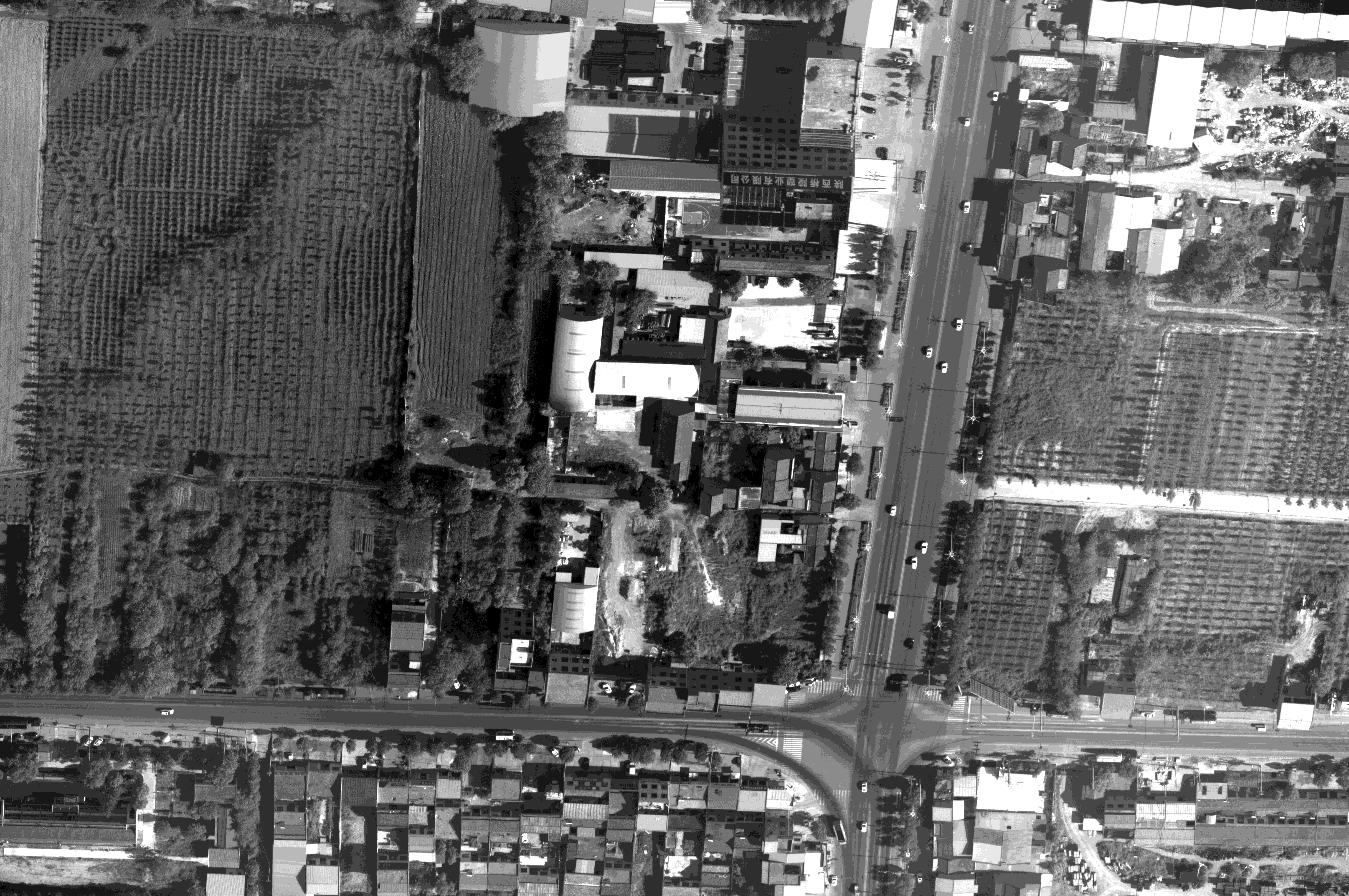

Supplement: S1 Data — (ZIP) [file pone.0274773.s001.zip › dataset/image/17-2388.jpg]

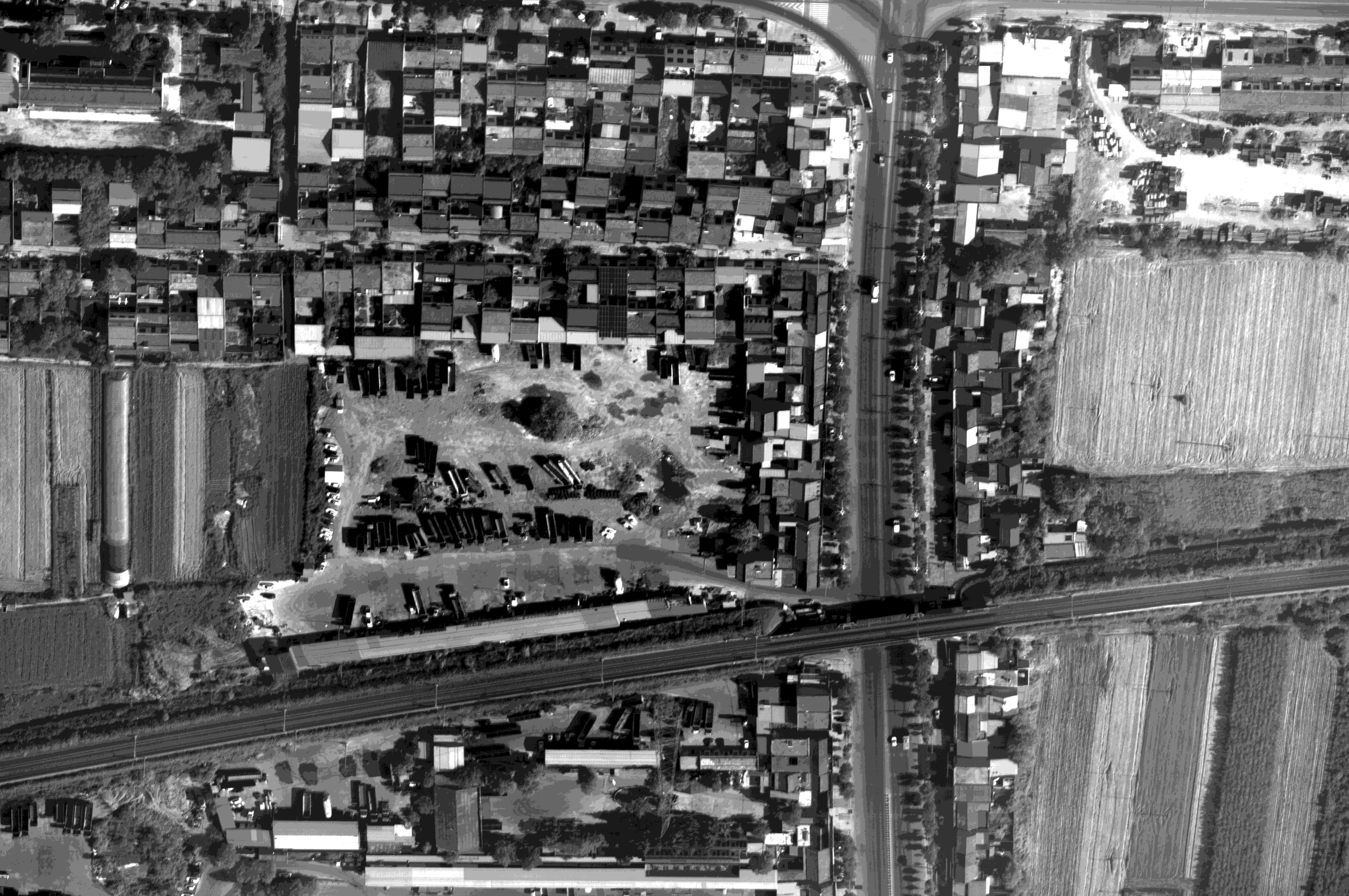

Supplement: S1 Data — (ZIP) [file pone.0274773.s001.zip › dataset/image/17-2389.jpg]

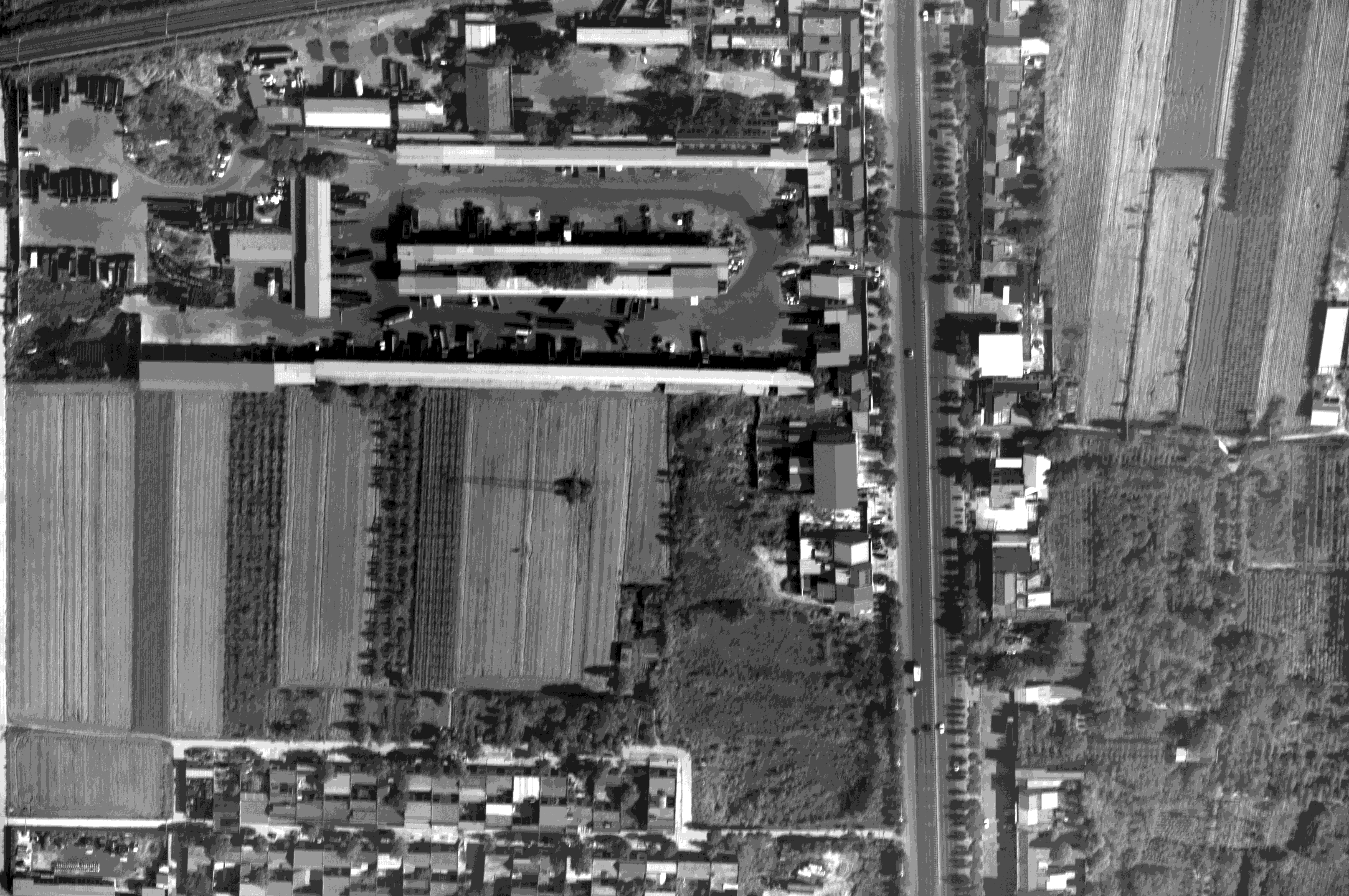

Supplement: S1 Data — (ZIP) [file pone.0274773.s001.zip › dataset/image/17-2390.jpg]

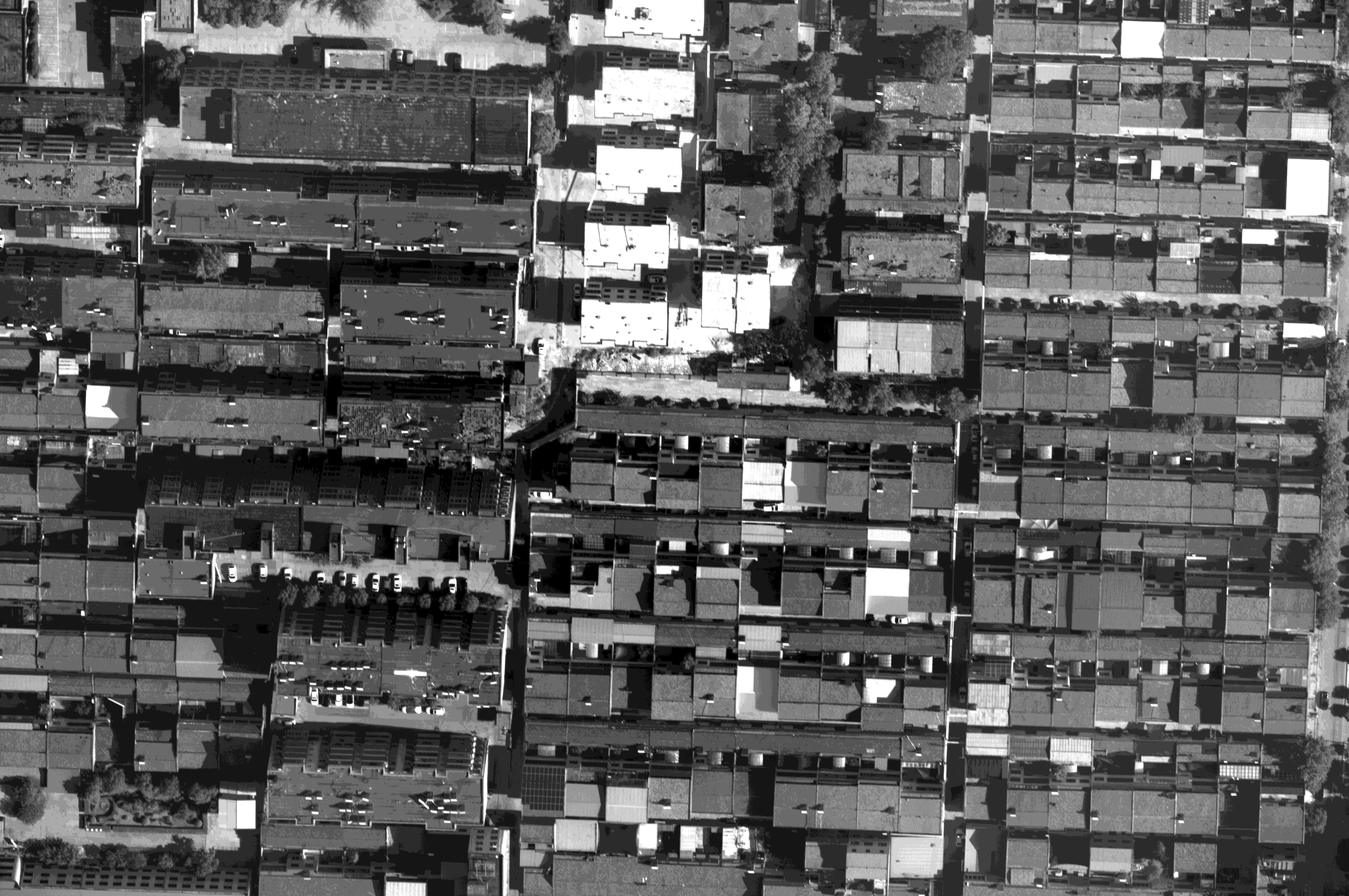

Supplement: S1 Data — (ZIP) [file pone.0274773.s001.zip › dataset/image/17-2391.jpg]

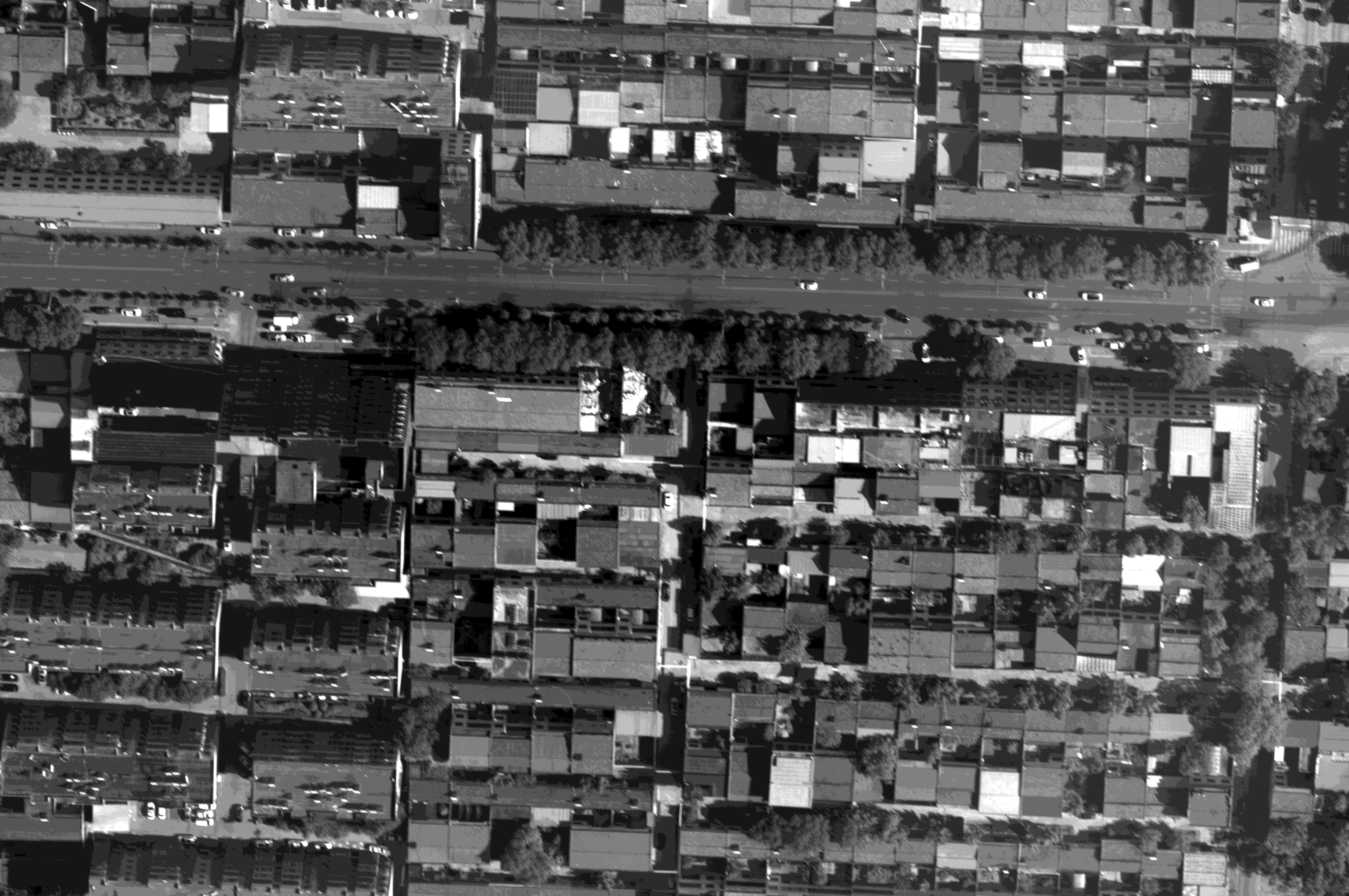

Supplement: S1 Data — (ZIP) [file pone.0274773.s001.zip › dataset/image/17-2392.jpg]

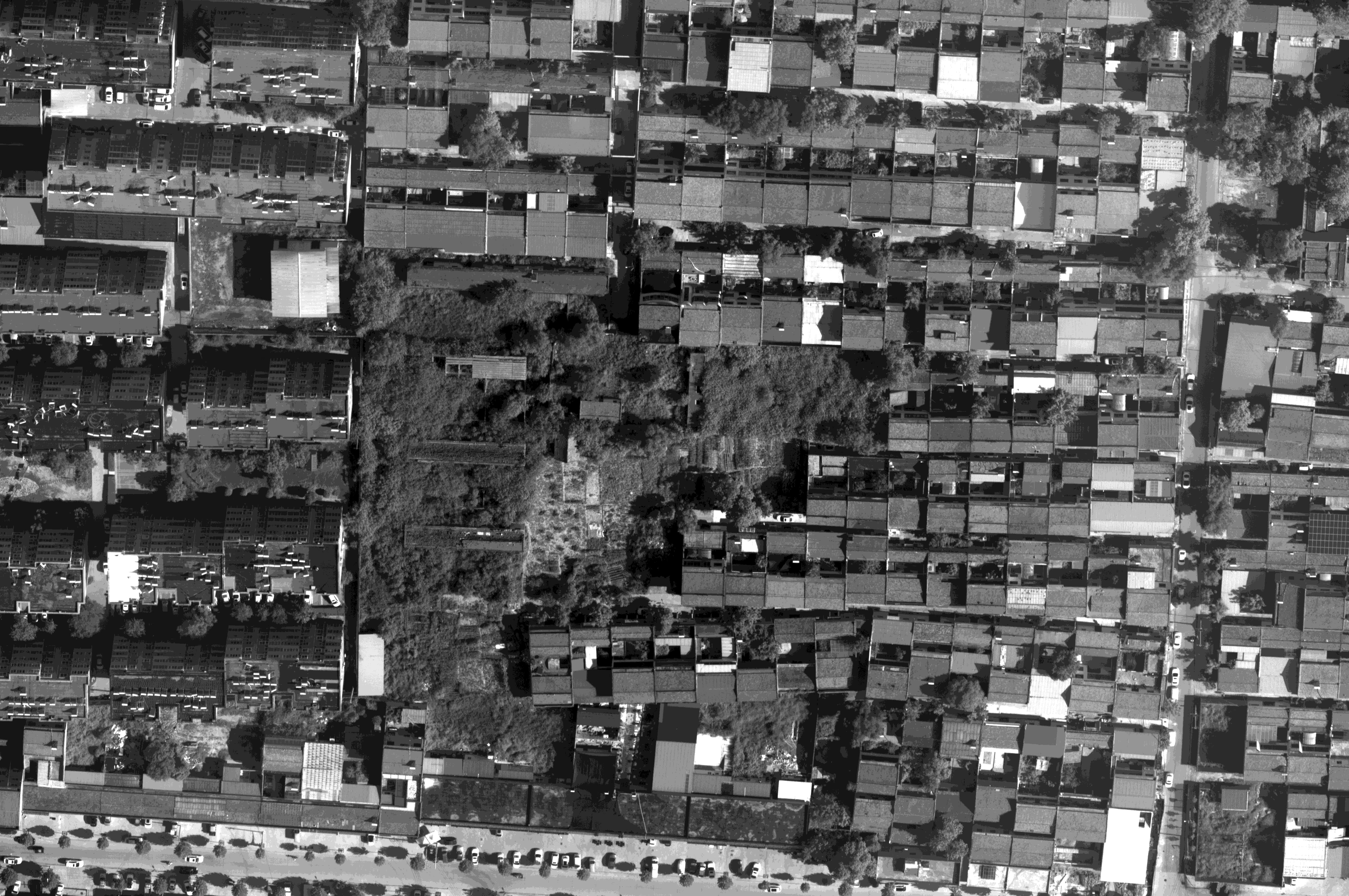

Supplement: S1 Data — (ZIP) [file pone.0274773.s001.zip › dataset/image/17-2393.jpg]

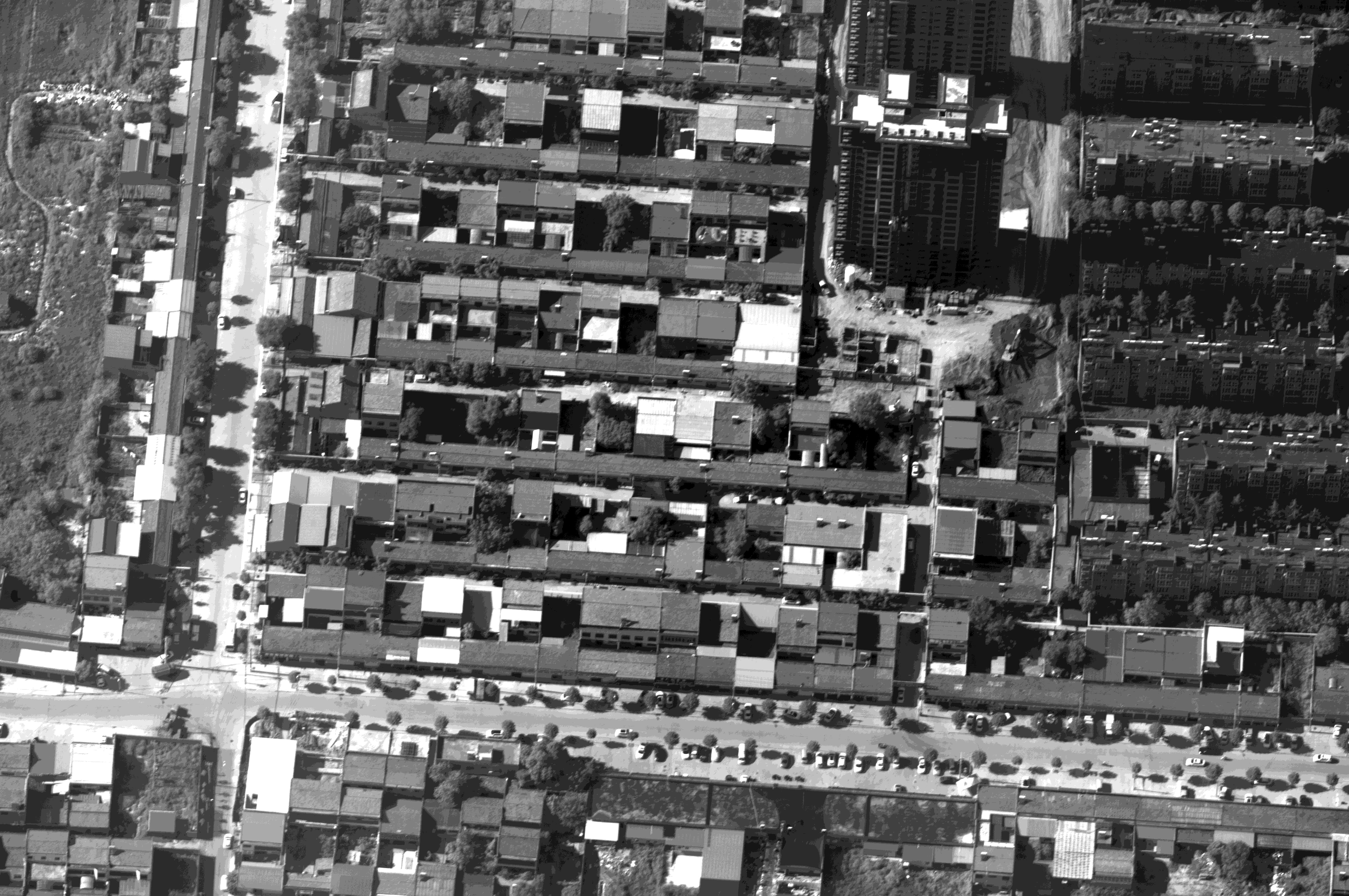

Supplement: S1 Data — (ZIP) [file pone.0274773.s001.zip › dataset/image/17-2394.jpg]

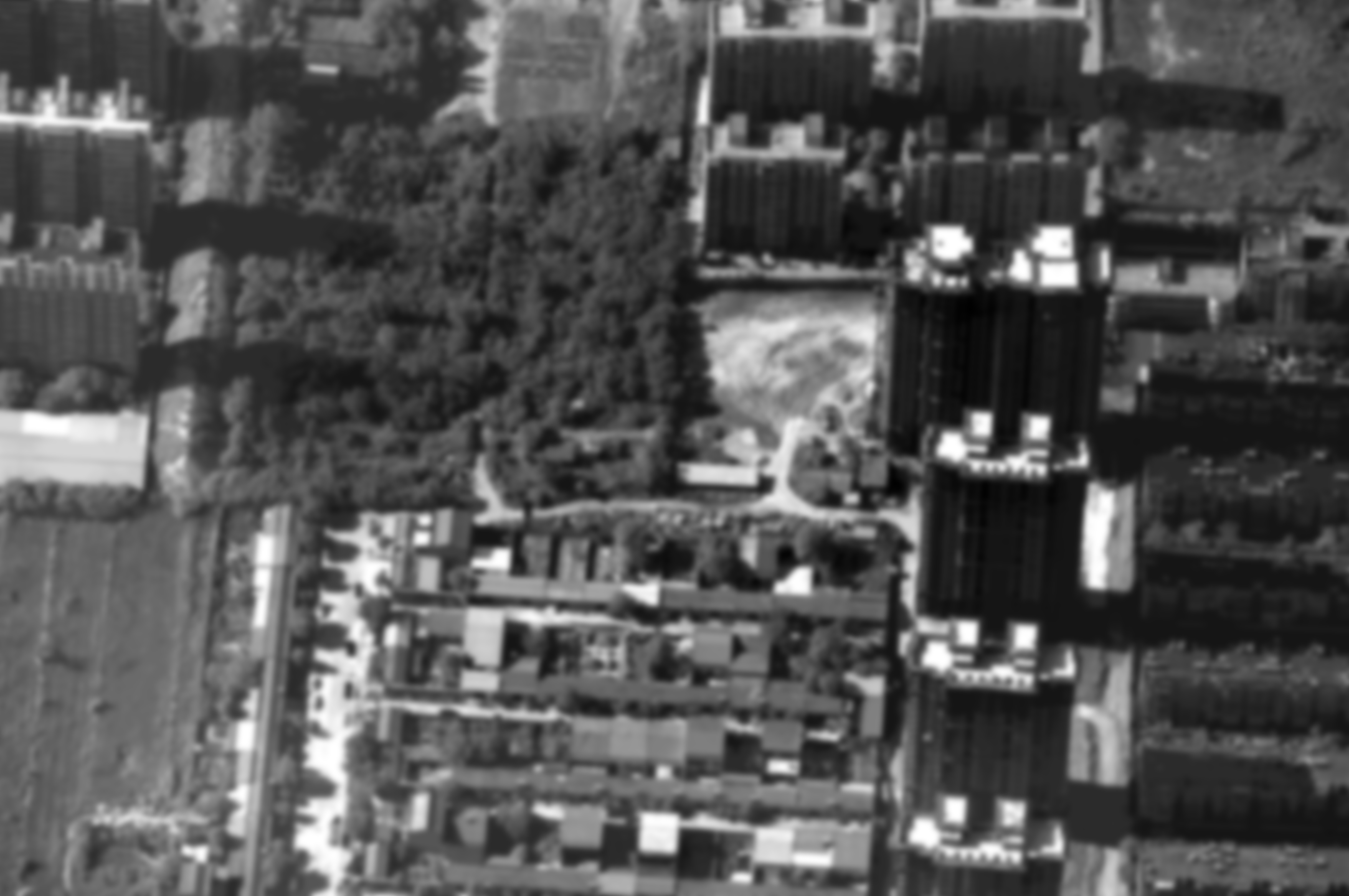

Supplement: S1 Data — (ZIP) [file pone.0274773.s001.zip › dataset/image/17-2395.jpg]

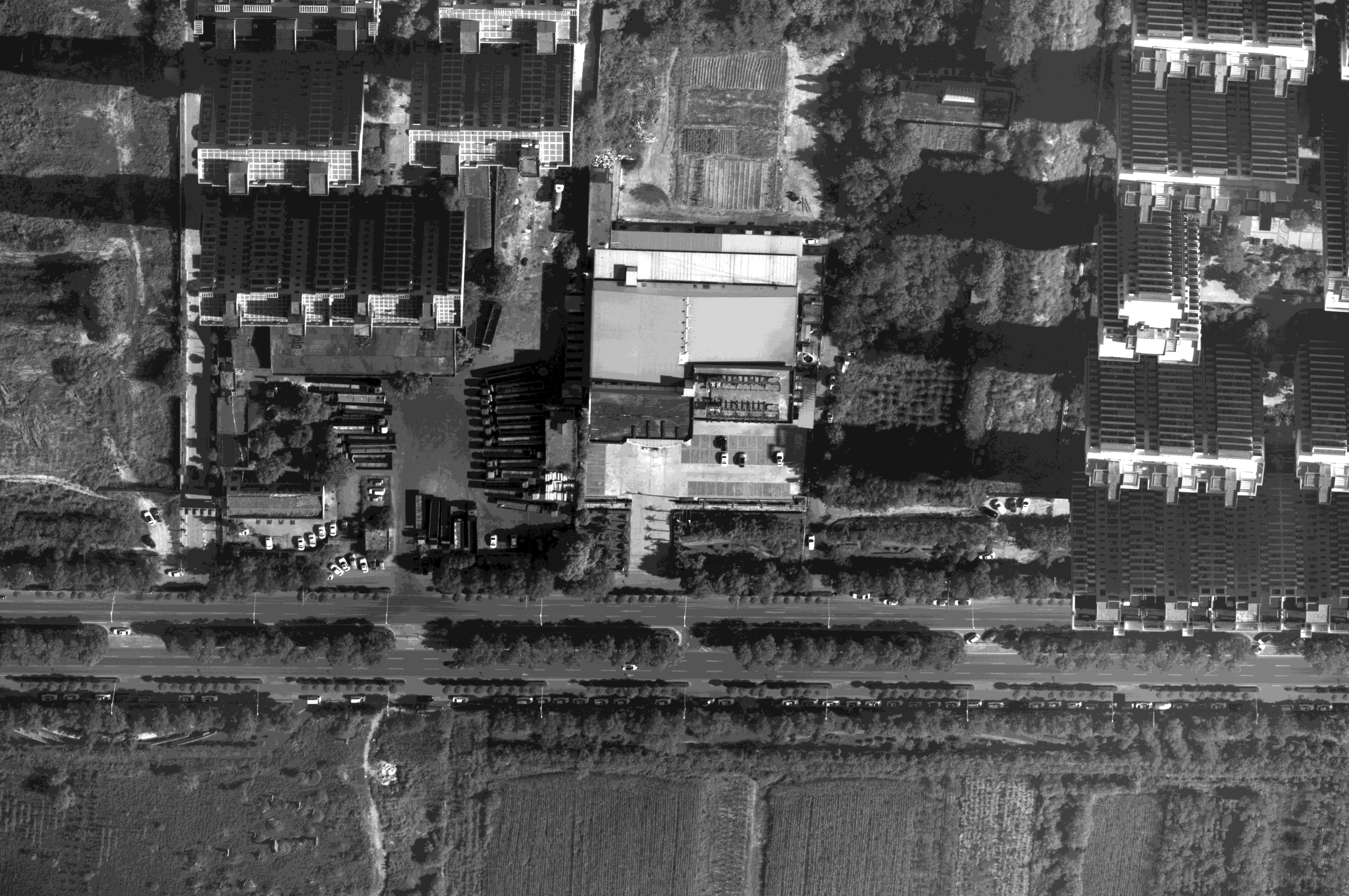

Supplement: S1 Data — (ZIP) [file pone.0274773.s001.zip › dataset/image/17-2396.jpg]

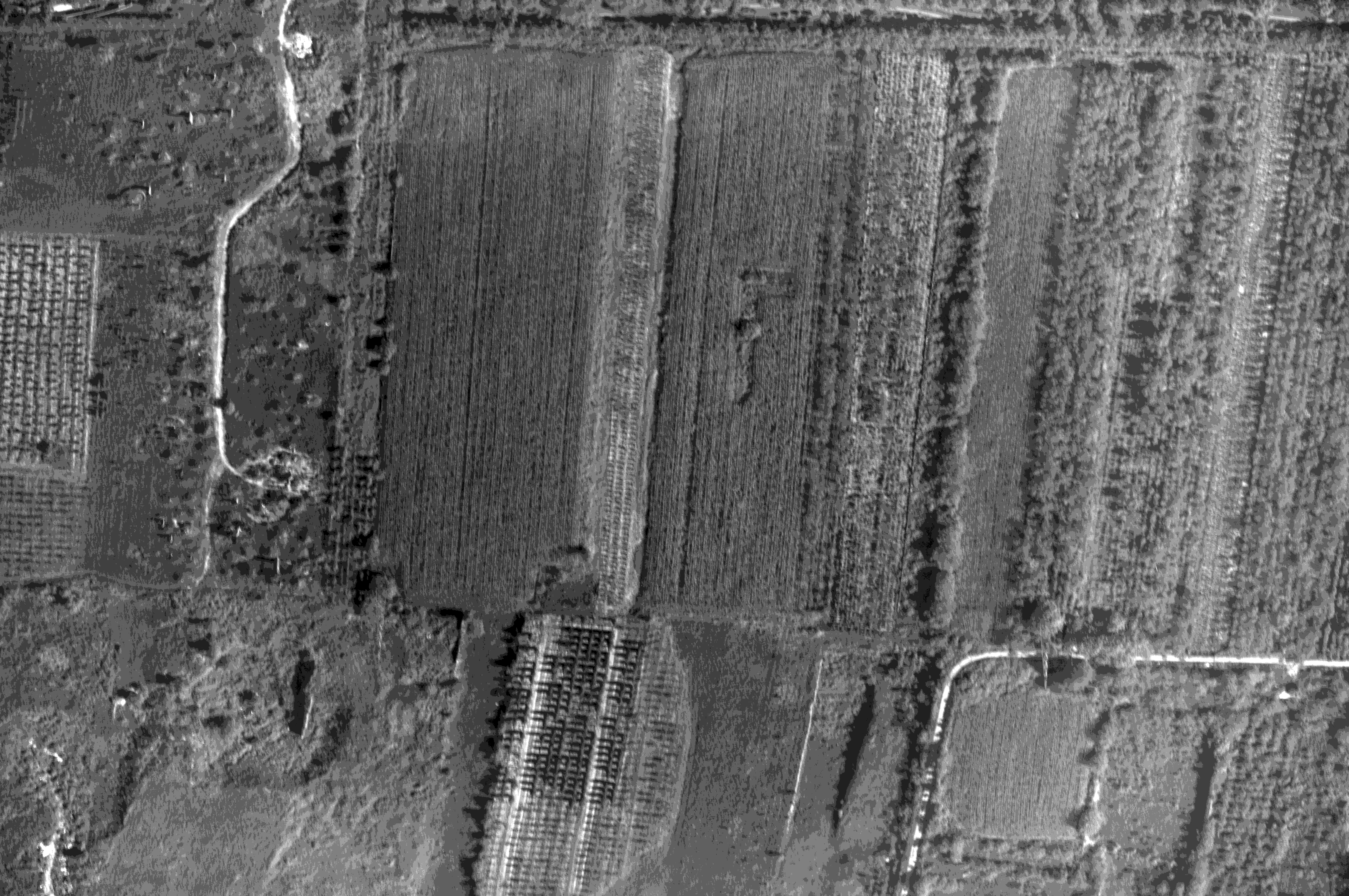

Supplement: S1 Data — (ZIP) [file pone.0274773.s001.zip › dataset/image/17-2397.jpg]

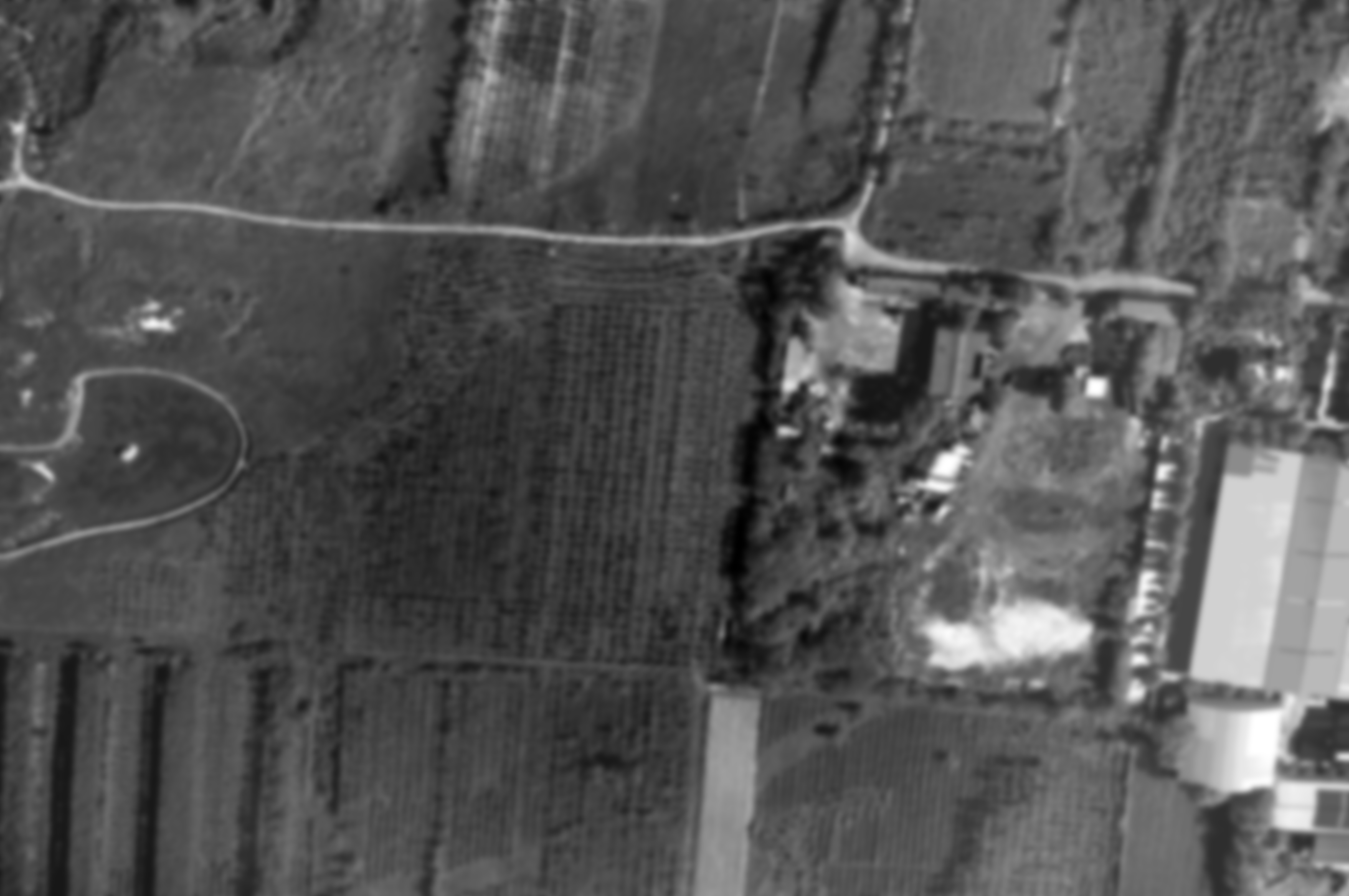

Supplement: S1 Data — (ZIP) [file pone.0274773.s001.zip › dataset/image/17-2398.jpg]

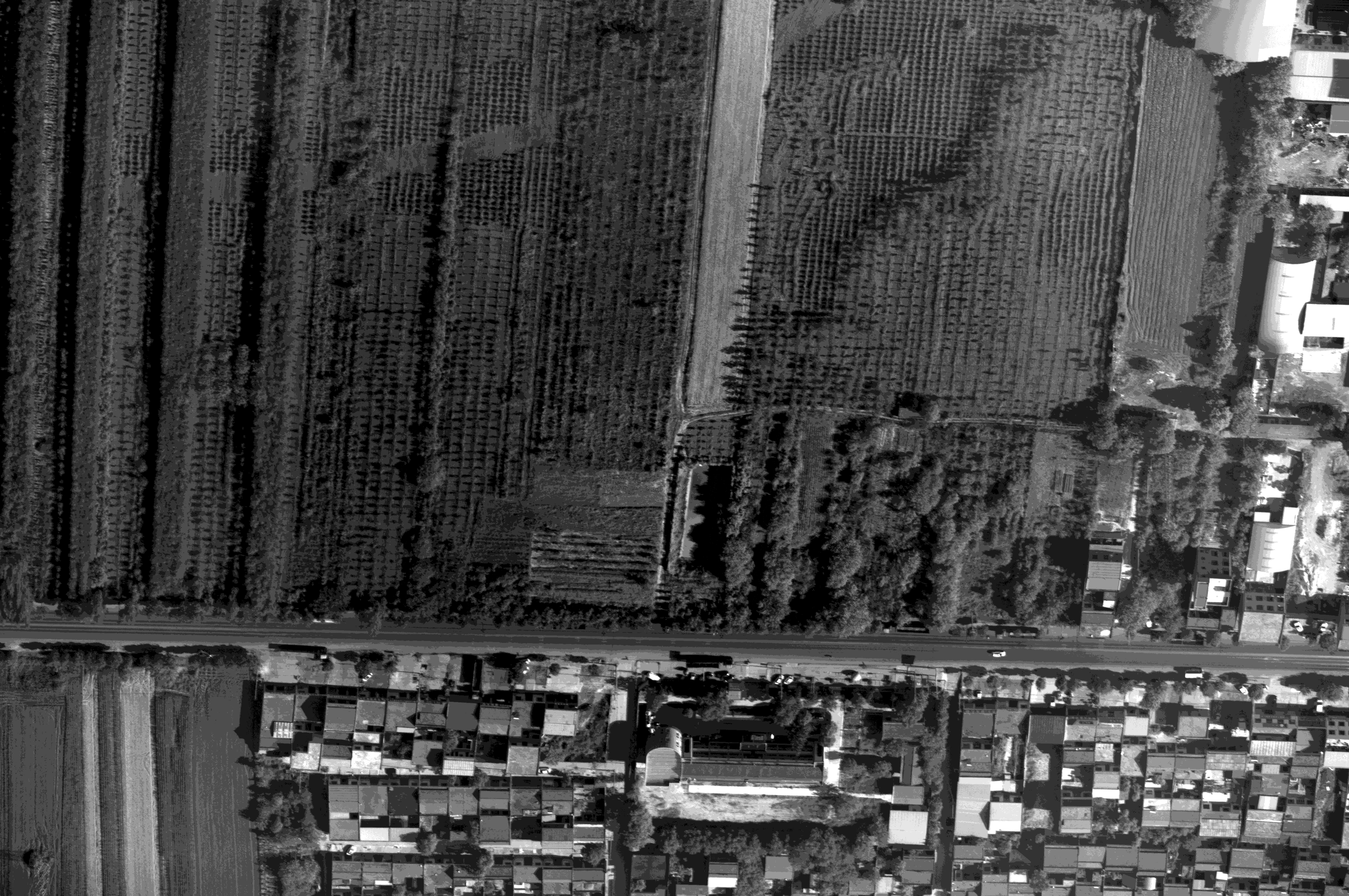

Supplement: S1 Data — (ZIP) [file pone.0274773.s001.zip › dataset/image/17-2399.jpg]

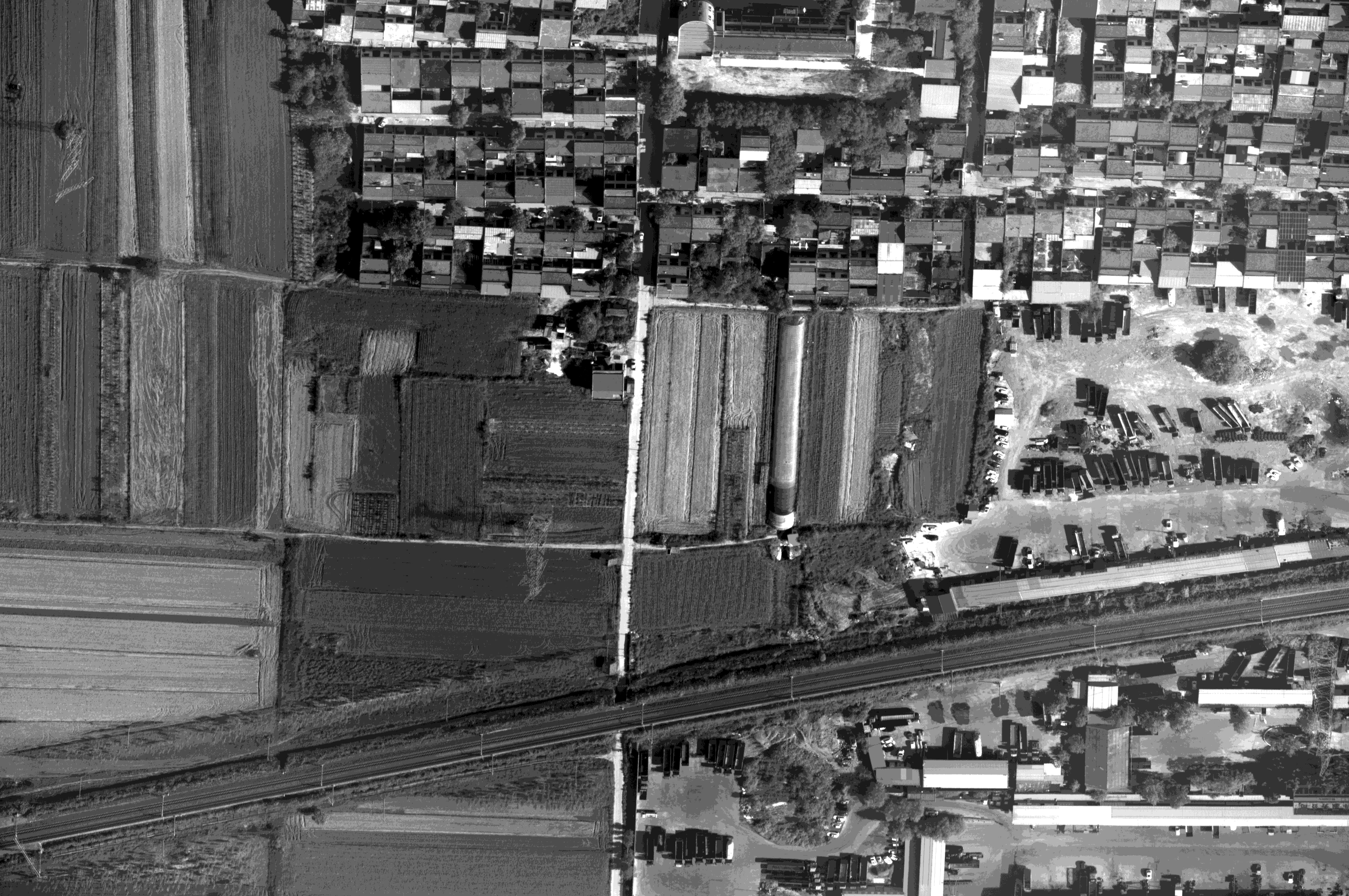

Supplement: S1 Data — (ZIP) [file pone.0274773.s001.zip › dataset/image/17-2400.jpg]
